# Development of amoebic liver abscess in early pregnancy years after initial amoebic exposure: a case report

**DOI:** 10.1186/s12876-020-01567-7

**Published:** 2020-12-14

**Authors:** Rainer W. J. Kaiser, Julian Allgeier, Alexander B. Philipp, Julia Mayerle, Camilla Rothe, Claudia Wallrauch, Mark op den Winkel

**Affiliations:** 1 Department of Medicine I, LMU Klinikum, Ludwig-Maximilians-University, Munich, Germany; 2 DZHK (German Centre for Cardiovascular Research), Partner Site Munich Heart Alliance, Munich, Germany; 3 Department of Medicine II, LMU Klinikum, Ludwig-Maximilians-University, Munich, Germany; 4 Department of Tropical Medicine, LMU Klinikum, Ludwig-Maximilians-University, Munich, Germany

**Keywords:** Entamoeba histolytica, Amoebic liver abscess, Pregnancy, Paromomycin, Embryotoxicity, Case report

## Abstract

**Background:**

Infection with *Entamoeba histolytica* and associated complications are relatively rare in developed countries. The overall low prevalence in the Western world as well as the possibly prolonged latency period between infection with the causing pathogen and onset of clinical symptoms may delay diagnosis of and adequate treatment for amoebiasis. Amoebic liver abscess (ALA) is the most common extraintestinal manifestation of invasive amoebiasis. Pregnancy has been described as a risk factor for development of invasive amoebiasis and management of these patients is especially complex.

**Case presentation:**

A 30-year-old Caucasian woman in early pregnancy presented to our emergency department with abdominal pain alongside elevated inflammatory markers and liver function tests. Travel history revealed multiple journeys to tropic and subtropic regions during the past decade and a prolonged episode of intermittently bloody diarrhea during a five month stay in Indonesia seven years prior to admission. Sonographic and magnetic resonance imaging revealed a 5 × 4 cm hepatic abscess. After ultrasound-guided transcutaneous liver drainage, both abscess fluids and blood cultures showed neither bacterial growth nor microscopic signs of parasitic disease. Serological testing confirmed an infection with *Entamoeba histolytica*, which was treated with metronidazole, followed by eradication therapy with paromomycin. Subsequent clinical, laboratory and imaging follow-up exams showed regression of the ALA. In addition, the pregnancy completed without complications and a healthy baby boy was born 7 months after termination of treatment.

**Conclusions:**

This case of invasive amoebiasis in early pregnancy outside of endemic regions and several years after exposure demonstrates the importance of broad differential diagnostics in the context of liver abscesses. The complex interdisciplinary decisions regarding the choice of imaging techniques as well as interventional and antibiotic therapy in the context of pregnancy are discussed. Furthermore, we present possible explanations for pregnancy as a risk factor for an invasive course of amoebiasis.

## Background

Amoebiasis, which is caused by infection with the anaerobic protozoan *Entamoeba histolytica* and other *Entamoeba* species [1], is recognized as the fourth-leading cause of death by parasite infection worldwide [2]. Annually, around 50 million patients develop symptoms of amoebic dysentery or extraintestinal disease with an estimated 50,000 to 100,000 deaths per year caused by complicated infection of this unique pathogen [1–6]. With humans and probably human-like primates as the only natural hosts, the sources of infection are water contaminated with *Entamoeba* cysts or—more rarely—fecal–oral contacts [1, 7]. Most developing countries with poor sanitation standards are endemic for the disease, while the majority of cases in developed countries of the Western world arises from immigration and patients traveling to endemic areas. The prevalence is higher in young men compared to women, patients on immunosuppressants as well as HIV-positive patients [1, 3, 4]. Of note, pregnancy is a known risk factor for development of invasive amoebiasis [1, 8, 9]. Few epidemiological data regarding the prevalence of *E. histolytica* in Europe and North America exist; however, studies investigating infectious travel-related gastrointestinal disorders have reported that amoebiasis is the identified pathogen in 1.4% of cases in Europe [10].

In the majority of amoebiasis cases (> 90%), infection with *Entamoeba* species, especially the non-pathogenic commensal *E. dispar*, is asymptomatic or self-limiting [1, 3]. However, around 10% of patients develop invasive amoebiasis, with the majority of patients suffering from amoebic dysentery or colitis. Within approximately 8–20 (median 12) weeks after exposure, patients report the subacute onset of typical symptoms including abdominal pain, fever and diarrhea with bloody and mucous stools [4]. One in 10 patients with invasive amoebiasis will develop extraintestinal disease, with amoebic liver abscesses (ALA) being the most common form; these patients typically present with increasing abdominal pain and tenderness in the right upper quadrant as well as fever over the course of a few days [1, 3, 11]. Apart from ALA, there are reports of cardiac, pleuropulmonary, cerebral as well as urogenital and dermatological complications secondary to amoebiasis [12–15].

In the case of suspected amoebic dysentery, direct microscopy of stool samples should be performed to detect the active form of *Entamoeba* species, the so-called trophozoite. In developed countries, serological testing (e.g. ELISA, immunofluorescence testing) remains the standard of care for securing the correct diagnosis [16]. Diagnosis of amoebic liver abscess is usually supported by imaging studies, namely abdominal ultrasound, contrast-enhanced ultrasound (CEUS)—where available—and high-resolution computed tomography to detect local extent of disease as well as infection-associated complications [16].

## Case presentation

**Fig. 1:**
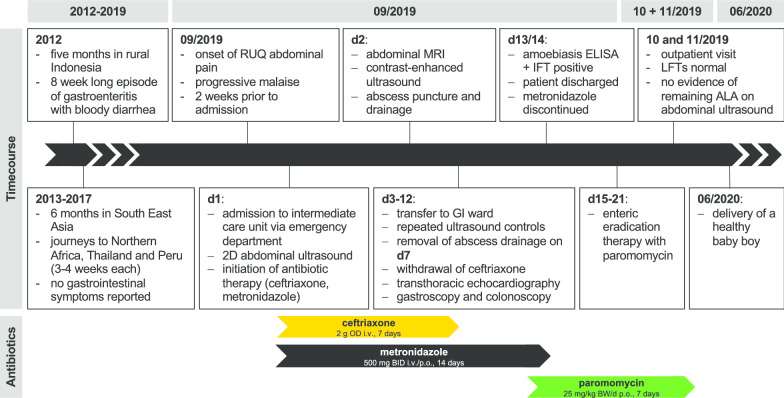
Timecourse of the patient’s travel history as well as the pre-, intra- and post-hospital course of treatment

A 30-year-old female Austrian patient was admitted to our emergency department with steadily worsening abdominal pain in the right upper quadrant, which had started about a week earlier. In addition, the patient suffered from nausea, headache and progressive malaise. The patient stated to be pregnant (week 5 + 3), which was confirmed by beta-HCG testing. Neither pre-existing medical conditions nor intake of any medication were reported. The patient had frequently traveled abroad during the previous decade, including prolonged journeys to South East Asia, northern Africa as well as South America (Fig. 1). Indeed, during a five month stay in Indonesia seven years before onset of symptoms, the patient reported to have suffered from an eight week-long episode of intermittently bloody diarrhea.

On clinical exam, the afebrile patient’s vital signs were stable. Abdominal tenderness was found in the right upper quadrant.

Laboratory tests revealed elevated levels of leukocyte counts (12 G/l, normal range [NR] 3.90–9.80 G/l), C-reactive protein (CRP, 25.3 mg/dl, NR 0.5 mg/dl) as well as pathologic values of alkaline phosphatase (AP, 305 U/l, NR 40–130 U/l) and gamma glutamyl transferase (GGT, 142 U/l, NR 59 U/l), while bilirubin levels were within normal range.

**Fig. 2:**
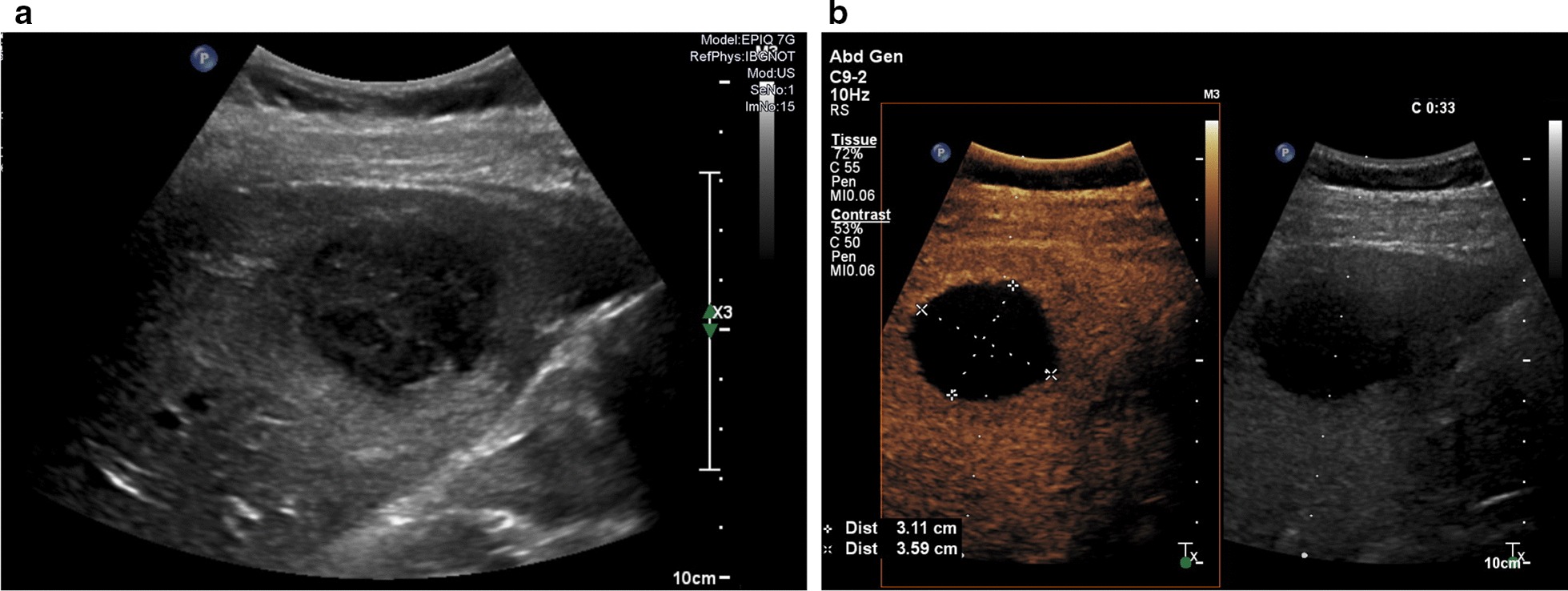
**a** 2D abdominal ultrasound performed on the day of presentation shows a hypoechogenic, inhomogenous and approximately 4 × 5 cm large lesion in hepatic segment V. **b** Contrast-enhanced ultrasound of the liver reveals peripheral hypervascularization of the lesion in segment V without central uptake of contrast, a finding consistent with liver abscess

**Fig. 3:**
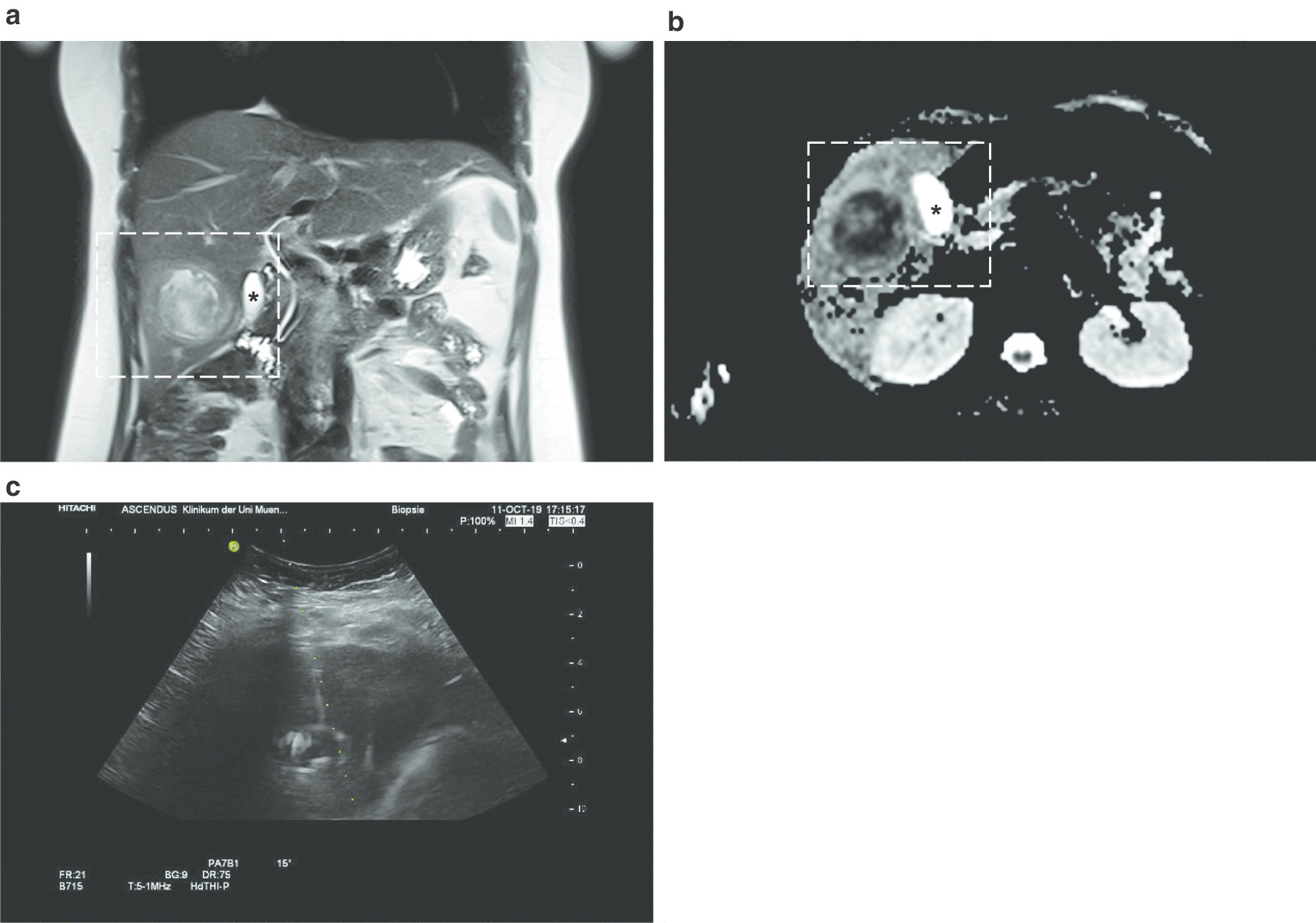
**a** Coronary MRI image of the liver without contrast reveals a T1-hypointense hepatic lesion, adjacent to the gall blader (indicated with *). **b** Axial MRI sequences showing diffusion restriction to the center of the lesion (* = gall bladder). **c** 2D sonography after successful transhepatic puncture and drainage. The green dots depict the direction of puncture, the hyperechogenic reflexes within the lesion are caused by the drainage

Ultrasound studies excluded cholestasis but showed a 5 × 4 cm hypoechogenic mass in segment V of the right liver lobe (Fig. 2a). CEUS revealed no central vascularization of the lesion, but a ring-like hypervascularization around the mass, consistent with a hepatic abscess (Fig. 2b). We did not perform computed tomography in light of the patient’s young age and pregnancy-status. Instead, MRI without contrast medium was performed, which showed a T1-hypo—(Fig. 3a) and T2-hyperintense hepatic lesion of 4.6 cm in diameter with both solid and fluid proportions, suspected septa within the lesion as well as restricted diffusion (Fig. 3b). The radiological differential diagnoses at this point included hepatic abscess, ruptured hydatid cyst and—though less likely—a necrotizing malignant tumor.

Considering the patient’s travel history, we extended serological testing for infectious diseases. *Echinococcus* spp. and other parasites as well as tuberculosis and HIV were ruled out. A malignant disease appeared unlikely making hepatic abscess formation the most likely differential diagnosis. Without knowing the causing pathogen, antibiotic therapy was initiated with a dual regimen of metronidazole (500 mg i.v., twice daily) and ceftriaxone (2 g i.v. once daily).

Aiming at fast-tracking the diagnostic and therapeutic process in light of the patient’s pregnancy and high CRP-values, ultrasound-guided abscess puncture with placement of a 10 french (F) drainage was performed (Fig. 3C, Additional file 1: Video 1). The drained fluid appeared brownish in color and contained bloody streaks (Additional file 2: Fig. S1A). Further fluid analysis showed a high amount of both neutrophil and eosinophil granulocytes; cultures from both the abscess and peripheral blood did not reveal any growth of bacteria, fungi or parasites. In addition, a PCR targeting prokaryotic 16S and fungal 18S/28S ribosomal RNA did not yield evidence of bacterial or fungal infection.

On antibiotic therapy and analgesic treatment, the patient’s condition improved. Laboratory tests confirmed reduction of inflammatory markers and LFTs, while control ultrasound studies showed shrinkage of the abscess. Six days after puncture, the drainage was removed.

Without having secured either focus or pathogen, we performed additional diagnostic studies to exclude extrahepatic manifestations of infection or an underlying malignancy. Transthoracic echocardiography was unremarkable. Gastroscopy and colonoscopy revealed a normal macroscopic aspect of the mucosa and histological examination excluded inflammatory bowel disease, active infectious colitis or signs of malignancy.

Shortly after, positive amoebic serology was confirmed by both amoebiasis-EIA (enzyme immunoassay; 100 U, NR < 10 U) and amoebiasis-IFT (immunofluorescence testing; 1:128, NR < 1:64).

Ceftriaxone was withdrawn, and metronidazole administration was continued until a total treatment-duration of 14 days (Fig. 1).

Subsequently, a one-week-regimen of the intraluminal acting aminoglycoside paromomycin was administered (25 mg/kg BW/d) after excluding macro- and microscopic signs of disturbed gut-blood barrier (e.g. acute inflammation) by endoscopy. This decision was based on thorough interdisciplinary discussion with clinical pharmacology, infectious disease department as well as gynecology. In light of the long remaining time of pregnancy, administration of paromomycin to reduce the risk of recurrent invasive disease due to remaining intestinal amoeboid cysts was considered beneficial.

With continued clinical improvement, the patient was discharged.

During the follow-up visits to our hepatology and tropical medicine outpatient-clinic two and four weeks after discharge, the patient was in excellent condition. Laboratory tests showed almost complete normalization of LFTs, normal white blood counts and CRP levels as well as a concomitant rise in beta-HCG. Abdominal ultrasound revealed complete regression of the abscess and transvaginal ultrasound documented normal embryonic development. No adverse reactions to the antibiotic treatment were reported throughout pregnancy, and there were no clinical signs of relapsing amoebiasis. Seven months after discharge, a healthy child was delivered.

## Discussion and conclusion

*Entamoeba histolytica* is a pathogen which is isolated comparatively rarely from travelers returning from tropical/subtropical countries, nevertheless, one not to miss [17]. Concise taking of medical and travel history is key in identifying *E. histolytica* as the potential culprit. Clinical manifestation of intestinal or extraintestinal amoebiasis several years after exposure is rather uncommon, with most patients reporting typical GI-symptoms like bloody diarrhea and pan-abdominal pain 8–20 weeks after returning from endemic countries. However, overt manifestation of invasive amoebiasis years or even decades after traveling to endemic regions has been reported [9, 16, 18].

Most features of our case can be regarded as typical for ALA: Localization of a solitary lesion in the right liver lobe [19, 20], typical clinical, imaging and serology findings in a patient with relevant travel history to endemic countries. The complexity regarding its diagnosis and clinical management arises mainly from the low prevalence of this condition in our developed non-endemic country, the long latency period and above all, the concurrent pregnancy.

In our case, pregnancy might have served as a predisposing factor in reactivation of amoebic infection. The presence of a fetus requires an altered immunological response and results in a variety of immunomodulating processes with measurable changes in cellular and humoral immunity [21]. These processes cannot only alter the susceptibility to certain diseases but also increase the severity of the impact of the pathogen. While pregnancy cannot be regarded as a state of systemic immunosuppression, there are measurable changes both at the cellular and humoral level. This includes essential shifts in the makeup of the cellular immune response to pathogens and the self, for example from T-helper 1 (Th1)- to T-helper 2 (Th2)-lymphocytes, increasing B-cell activation and production of antibodies while decreasing cytotoxic T-cell responses. Humoral changes include decreased release of chemokines and growth factors such as tumor necrosis factor (TNF-), IFN-, interleukin (IL)-15, vascular endothelial growth factor A (VEGF-A), and chemokine (C–C motif) ligand 2 (CCL2) [22]. Epidemiological evidence has shown a tendency for pregnant women to be at risk for more severe course of disease for various viral, bacterial and fungal infections including influenza, herpes simplex, hepatitis E, listeriosis or coccidioidomycosis, yet there is little evidence that susceptibility is increased as well [21]. Immunological changes are also facilitated by increased production of steroid hormones, progesterone and estrogen as pregnancy takes its course. High progesterone levels also drive T helper cell differentiation towards the anti-inflammatory Th2-phenotype and secretion of anti-inflammatory cytokines including IL-4, IL-5, and IL-10; they also drive differentiation of macrophages to a state coined M2 phenotype, associated with healthy, full-term pregnancies. In contrast, M1 macrophage phenotype is associated with elevated secretion of IL-12 and TNF and associated with preterm birth. Similar roles are employed both by estradiol and estriol, where high concentrations are also associated with an anti-inflammatory phenotype in cellular immunity [22]. In addition, there is increasing epidemiological and experimental evidence on the role of testosterone in extraintestinal amoebiasis: ALA is more common in males (up to 9:1 male-to-female ratio in the age group of 20–40 year olds), and since testosterone plasma levels in females can double in the setting of pregnancy, its immunopathological effects on monocyte function further illustrate heightened susceptibility to a variety of clinical manifestations of amoebiasis [23]. Two murine models of ALA seem to indicate this increased male susceptibility is due to altered function of macrophage and natural killer (NK) cell populations in response to high testosterone levels [24, 25]. Containing *Entamoeba histolytica* is a task heavily relying on the inflammatory cellular response mediated by the M1 macrophages and TNF and IFN cytokine activity. Even after initial intestinal infection, colonization of *E. histolytica* can persist and result in reinfection and extraintestinal manifestation upon altered macrophage and cytokine activity [26, 27], which may have contributed to ALA development in our patient.

Imaging modalities need to be chosen with care in the pregnant patient. The American College of Obstetricians and Gynecologists (ACOG) guidelines recommend ultrasound and MRI as the modalities of choice [28]. In our setting, MRI without contrast was performed after initial ultrasound assessment; subsequently, the accessibility of the abscess enabled successful ultrasound-guided drainage. Thus, imaging with ionizing radiation could be avoided [29]. Given other circumstances, such as typical symptoms of intestinal amoebiasis including recent fever and bloody diarrhea as well as a patient presenting in or coming from a geographic region with higher prevalence of amoebiasis, imaging could be restricted to abdominal ultrasound followed by sole diagnostic puncture instead of placement of a drainage catheter. In our case, initial uncertainty about the nature of the hepatic lesion, the clinical symptoms including right upper quadrant abdominal pain as well as the high CRP-value in a pregnant patient justified the catheter drainage [1].

Aspiration or catheter drainage of ALA in addition to the antimicrobial therapy to hasten clinical resolution is not mandatory in uncomplicated ALA in the setting of rather small size (< 5 cm diameter) and no risk factors for abscess rupture (e. g., marginal abscess with limited amount of liver tissue around the abscess formation) [29]. The material aspirated from an ALA, mainly consisting of liquified liver tissue, is in itself of limited diagnostic value as trophozoites can only be microscopically detected in a minority of aspirates (< 20%). With varying sensitivity and specificity of both microscopic and immunological diagnostic tools, novel and promising PCR-based diagnostic methods have been developed that enable the fast rule-in or rule-out of a variety of parasites, including *E. histolytica* (as reviewed by Ryan et al*. *[30] and Madden et al*. *[31]). However, there are neither standardized PCR protocols nor commercially available detection kits, resulting in varying degrees of sensitivity and specificity of the often applied in-house protocols [32]. Further improvement of molecular diagnostic methods as well as enhanced accessibility in developing countries may facilitate diagnosis of invasive amoebiasis in general as well as ALA in particular [33].

Metronidazole and the structurally similar tinidazole are the therapeutic agents used for treatment of acute infection with *E. histolytica* [34]. Metronidazole is the treatment of choice for pregnant women [35]. Metronidazole is a US Food and Drug Administration (FDA) pregnancy category B drug, is generally well tolerated and although it crosses the placenta it seems to have no significant embryotoxic effects [36]. The initial situation with pending serology results required additional therapy with i.v. ceftriaxone. Differentiation between amoebic and bacterial liver abscess by clinical and imaging findings is not possible and a bacterial abscess may be caused by metronidazole-unresponsive bacteria such as *Enterococcus spp.* or *Klebsiella spp.*, which warrant the use of broad-spectrum antibiotics such as third generation cephalosporins [37]. The second anti-amoebic agent required specifically for intraluminal eradication of entamoebic cysts and therefore holistic treatment of amoebiasis is the aminoglycoside paromomycin, an FDA pregnancy category C drug. Paromomycin should not be given in pregnancy with concurrent severe (amoebic) colitis, because inflammation of the colonic mucosa could cause breakdown of the intestinal barrier and subsequent risk for systemic side effects. Paromomycin derives its systemic side-effect profile from its aminoglycosidic origin analogous to its systemically employed pharmacological siblings gentamicin and amikacin [38]. Gentamicin is known for its oto- and nephrotoxic side effects and characterized as a pregnancy category D drug by the FDA. Evidence indicates the possibility of these complications in the fetus, however, placental concentrations in the treatment of chorioamnionitis for example have been shown to be significantly lower than those in maternal blood, mediated by accelerated kidney function in pregnant patients [39]. Paromomycin is essential for intraluminal eradication of *E. histolytica* and there is no evidence supporting fetotoxicity [1, 3]. We considered a holistic treatment indispensable in our setting to prevent re-infection and further endangerment of the growing fetus. After consultation with gynecology, tropical medicine and clinical pharmacology, and after endoscopy confirmed the absence of mucosal damage, paromomycin was given. Eleven months after discharge, both patient and her newborn child remain well, with no evidence of any treatment-related side-effects.

## Limitations

With both the clinical diagnosis of liver abscess and a positive *E. histolytica* serology, the definition of confirmed amoebic liver abscess is met [40–42]. However, our case lacks additional direct evidence of the causing pathogen *E. histolytica* from abscess or stool samples by PCR; this might have sped up the definitive diagnosis as well as supported the need for intraluminal eradication therapy with paramomycin. The latter decision however was based on an individual and interdisciplinary assessment regarding the risk of *E. histolytica* recurrence during the long remaining time of pregnancy and given the possibility of a false negative *E. histoloytica* PCR [30]. The decision in this unique setting would not have been influenced by a stool-PCR test; indeed, recent German guidelines support the use of a luminal eradication agent regardless of the detection of amoeba in the intestine [37]. As mentioned above, different circumstances and a higher pre-test probability regarding the diagnosis of amoebic liver abscess may restrict applied diagnostics to ultrasound and diagnostic puncture without the need of more advanced imaging techniques like MRI and transcutaneous drainage.

To our knowledge, we present the first report of an extraintestinal invasive amoebiasis in early term pregnancy in western Europe, requiring an interdisciplinary approach and careful weighing of therapeutic options to effectively treat infection while maintaining safety for patient and fetus alike.

This case of invasive amoebiasis in early pregnancy outside of endemic regions and several years after exposure demonstrates the importance of broad differential diagnostics in the workup of a liver abscess including thorough investigation of the patient’s travel history. Amoebiasis represents a common travel-associated parasitic gastrointestinal infection in western Europe and north America, however, it remains an underestimated disease [5, 43]. Management of the less frequent invasive form of amoebiasis in such a unique clinical setting like pregnancy is a complex task. The limited availability of studied treatment options pose both diagnostic and therapeutic challenges, and adequate individualized management requires the close collaboration of various clinical departments [5, 43].

## Supplementary Information

**Additional file 1. Video 1:** Video clip of 2D sonography during transhepatic puncture and drainage (see Fig. 3C). A 10F drainage was placed. The hyperechogenic reflexes within the lesion are caused by the drainage.

**Additional file 2. Fig. S1:** Abscess aspirates after ultrasound-guided hepatic puncture with brownish color and bloody streaks. A 10F drainage was placed and flushed with 0.9% NS three times daily.

## Data Availability

Data sharing is not applicable to this article as no datasets were generated or analyzed in this study.

## Abbreviations

ACOG: American College of Obstetricians and Gynecologists
BW: Body weight
CCL: Chemokine (C–C motif) ligand
FDA: US Food and Drug Administration
HIV: Human immunodeficiency virus
Hb: Hemoglobin
IFN: Interferon
IL: Interleukin
i.v.: Intravenous
LFT: Liver function tests
NR: Normal range
NS: Normal saline
Spp.: Species
TB: Tuberculosis
Th1/2: T-helper cell 1/2
TNF-α: Tumor necrosis factor alpha
VEGF: Vascular endothelial growth factor
