# Supplementary figures and images for: Development of amoebic liver abscess in early pregnancy years after initial amoebic exposure: a case report
Source: BMC Gastroenterol. 2020 Dec 14;20:424. doi: 10.1186/s12876-020-01567-7 (PMC7734812; doi:10.1186/s12876-020-01567-7)

# Suppl. Figure 1

A

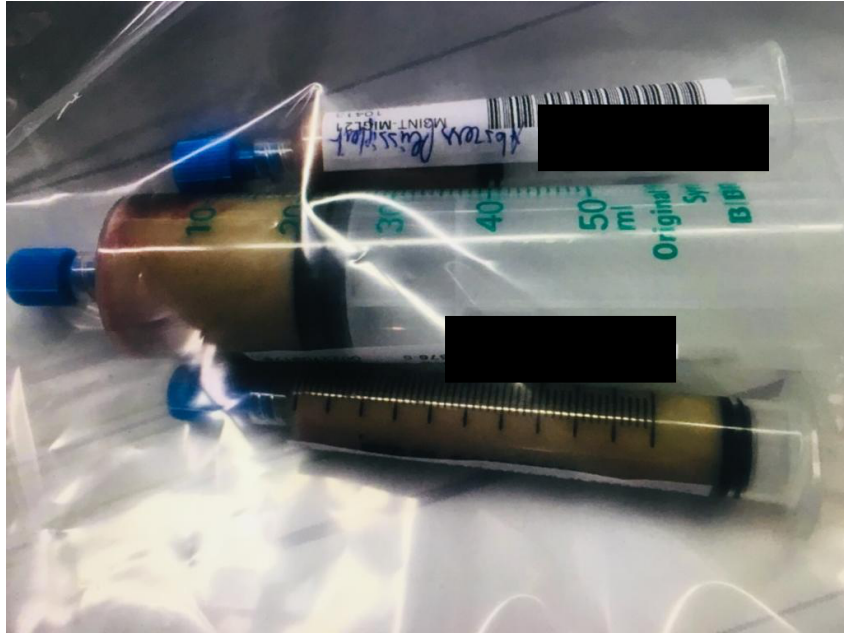

Supplement: Supplementary file 2 — Additional file 2. Fig. S1: Abscess aspirates after ultrasound-guided hepatic puncture with brownish color and bloody streaks. A 10F drainage was placed and flushed with 0.9% NS three times daily. [file 12876_2020_1567_MOESM2_ESM.pdf]
